# Comparison of the Immunogenicities and Cross-Lineage Efficacies of Live Attenuated Peste des Petits Ruminants Virus Vaccines PPRV/Nigeria/75/1 and PPRV/Sungri/96

**DOI:** 10.1128/JVI.01471-18

**Published:** 2018-11-27

**Authors:** Sophia Hodgson, Katy Moffat, Holly Hill, John T. Flannery, Simon P. Graham, Michael D. Baron, Karin E. Darpel

**Affiliations:** a The Pirbright Institute, Pirbright, Surrey, UK; b School of Veterinary Medicine, University of Surrey, Guildford, Surrey, UK; University of North Carolina at Chapel Hill

**Keywords:** immune response, livestock disease, morbillivirus, protection, vaccines

## Abstract

Peste des petits ruminants (PPR) is a severe disease of goats and sheep that is widespread in Africa, the Middle East, and Asia. Several effective vaccines exist for the disease, based on attenuated strains of the virus (PPRV) that causes PPR. While the efficacy of these vaccines has been established by use in the field, the nature of the protective immune response has not been determined. In addition, while the vaccine derived from PPRV/Nigeria/75/1 (N75) is used in many countries, those developed in India have never been tested for their efficacy outside that country. We have studied the immune response in goats to vaccination with either N75 or the main Indian vaccine, which is based on isolate PPRV/India/Sungri/96 (S96). In addition, we compared the ability of these two vaccines, in parallel, to protect animals against challenge with pathogenic viruses from the four known genetic lineages of PPRV, representing viruses from different parts of Africa, as well as Asia. These studies showed that, while N75 elicited a stronger antibody response than S96, as measured by both enzyme-linked immunosorbent assay and virus neutralization, S96 resulted in more pronounced cellular immune responses, as measured by virus antigen-induced proliferation and interferon gamma production. While both vaccines induced comparable numbers of PPRV-specific CD8^+^ T cells, S96 induced a higher number of CD4^+^ T cells specifically responding to virus. Despite these quantitative and qualitative differences in the immune responses following vaccination, both vaccines gave complete clinical protection against challenge with all four lineages of PPRV.

**IMPORTANCE** Despite the widespread use of live attenuated PPRV vaccines, this is the first systematic analysis of the immune response elicited in small ruminants. These data will help in the establishment of the immunological determinants of protection, an important step in the development of new vaccines, especially DIVA vaccines using alternative vaccination vectors. This study is also the first controlled test of the ability of the two major vaccines used against virulent PPRV strains from all genetic lineages of the virus, showing conclusively the complete cross-protective ability of these vaccines.

## Abstract (precis)

Despite the widespread use of live attenuated PPRV vaccines, this is the first systematic analysis of the immune response elicited in small ruminants. These data will help in the establishment of the immunological determinants of protection, an important step in the development of new vaccines, especially DIVA vaccines using alternative vaccination vectors. This study is also the first controlled test of the ability of the two major vaccines used against virulent PPRV strains from all genetic lineages of the virus, showing conclusively the complete cross-protective ability of these vaccines.

## INTRODUCTION

Peste des petits ruminants virus (PPRV) causes a severe disease of sheep and goats and has been spreading extensively over the past two decades; it is now found widely distributed through large parts of Africa, the Middle East, and Asia, posing an increasing threat to poor livestock keepers, primarily in developing countries (1–4). PPRV is a negative-strand RNA virus, a paramyxovirus of the genus *Morbillivirus*, related to the human pathogen measles virus (MV), as well as other animal pathogens such as canine distemper virus and the now-eradicated rinderpest virus (RPV). Control of PPR has recently become a major international goal, marked by the adoption in 2014 of a resolution by the World Organization for Animal Health (OIE) to establish a control program with a view to eventual eradication of the disease (5).

**FIG 1:**
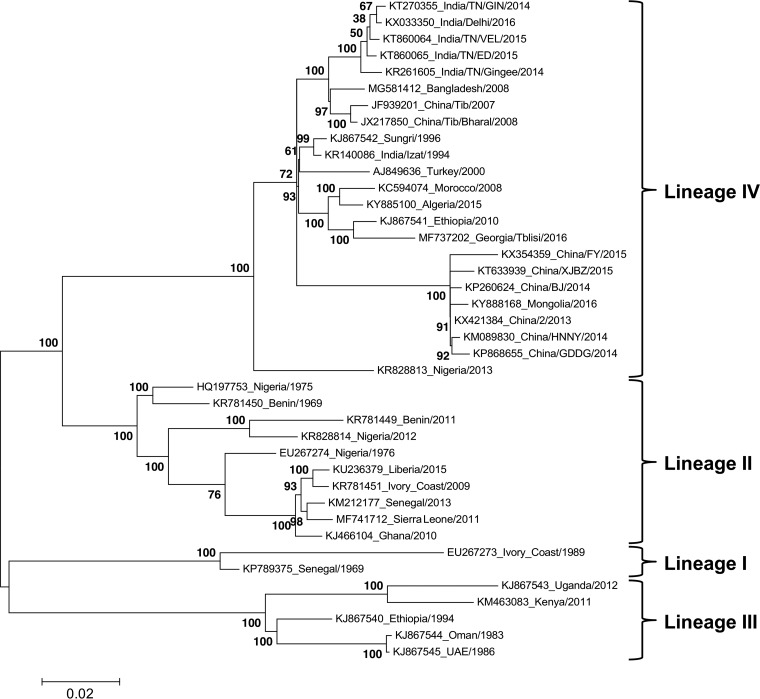
Phylogenetic tree of PPRV showing lineages I to IV. A phylogenetic tree showing the genetic distances between the available full-length PPRV genomes was calculated as described in Materials and Methods using MEGA6. The tree with the highest log likelihood is shown, with the percentage of trees in which the associated taxa clustered together in the bootstrap (500 replicates) shown next to the branches. The tree is drawn to scale, with branch lengths measured in the number of substitutions per site; the scale bar shows 0.02 substitutions per site. The labels at the ends of the branches show the accession number of the genome sequence and the country and year of the virus’s isolation. The clades considered as lineages I to IV are shown on the right.

As with other morbilliviruses, such as MV and RPV, there is no evidence of serotypes within PPRV, although sequence-based phylogenetic analysis has divided the known isolates into 4 genetic lineages (6, 7), with lineages I to III found in different parts of sub-Saharan Africa, while all Asian and almost all Middle Eastern isolates belong to lineage IV. The genetic distances between the lineages are relatively small, as illustrated by the phylogenetic tree shown in Fig. 1, which is based on the available full-length genome sequences (after removing all but one from each group of very similar sequences). Although PPR was first described as a unique disease in West Africa (8) and PPRV was first recognized as a separate virus in samples from East Africa (9), the virus probably originates in Asia (10), as did RPV, both viruses showing the same pattern of a single lineage covering Asia and the Middle East, with separate lineages appearing in Africa. In recent years, lineage IV viruses have also begun to appear in sub-Saharan Africa (11); an example of such an African lineage IV virus can be seen in PPRV/Nigeria/2013 in Fig. 1. In general, lineage II is the most similar to lineage IV, with lineages I and III forming a separate subgroup (Fig. 1); lineage I viruses are now uncommon and appear to be being replaced in West Africa by lineage II and lineage IV viruses.

Disease control is mostly achieved through the use of clinical or laboratory-based diagnosis coupled with vaccination and/or slaughter. All the vaccines currently in use are live attenuated strains of PPRV (12, 13), the two most commonly used being derived from PPRV/Nigeria/75/1 (12) (a lineage II isolate) or PPRV/India/Sungri/96 (14) (a lineage IV isolate). The Nigeria/75 (N75) vaccine is used in most countries outside India, while the Sungri/96 (S96) vaccine is used only in India. While both vaccines have provided lasting protection in field use, there has been no explicit testing of either vaccine against the full range of existing wild-type viruses. In particular, the S96 vaccine has only been shown experimentally to protect against two Indian isolates (15) and has been tested for neutralization only of itself (15, 16). The N75 vaccine has been shown, in different studies, to protect against virus isolates of all four lineages (12, 17–19), but the serum neutralization titer was again determined only against the vaccine itself, and we lack information on cell-mediated (T cell) responses in sheep/goats treated with either vaccine. This comparative lack of information has led to some workers suggesting a need for further vaccine strains to be developed based on local isolates (see, for example, reference 20). We have therefore directly compared the protection given by these two vaccines against representative wild-type challenge viruses from all four lineages. Establishing their universal efficacy would not only prevent wasted effort in making further vaccine strains but also improve the flexibility of supply of vaccine during the global eradication campaign by showing that supplies of either vaccine can be used in any country needing to vaccinate its small ruminant population. In addition, there is little information as to the range of immune responses elicited by these vaccines, the primary test of vaccine efficacy being a functional one of protection against virulent challenge. We have therefore examined the antibody and T cell responses elicited by vaccination of goats with either N75 and S96, data which will provide the foundation for future work on establishing the basis of protection provided by these vaccines.

## RESULTS

In total, five rounds of studies were conducted, in each of which five animals were vaccinated with N75, five animals were vaccinated with S96, and two animals were left unvaccinated to validate the pathogenicity of the challenge virus used. Partial results from the first study carried out, using challenge with PPRV/Ivory Coast/89, have previously been published (21); these results are included here for completeness and to provide the maximum power to our analysis of the response to the vaccine. In each round of studies, the ten vaccinates and two control animals were infected with a representative isolate from lineage I, II, III, or IV at 28 days postvaccination (dpv) to assess protection; when it was observed that the challenge virus selected from lineage II (PPRV/Nigeria/76/1) gave only very mild disease, a further group of animals was vaccinated and challenged using a more virulent challenge virus from the same lineage (PPRV/Ghana/78). One N75 vaccinate in one study was removed due to an unrelated underlying health issue. While this gave a total of 24 or 25 animals vaccinated with, respectively, N75 or S96, limitations in obtained material or processing time required that certain antibody or cell-based assays were carried out using a representative number of samples. In all of the following data, the numbers of experimental animals on which the analysis is based are given in the respective figure legends.

### Antibody responses to vaccination.

**FIG 2:**
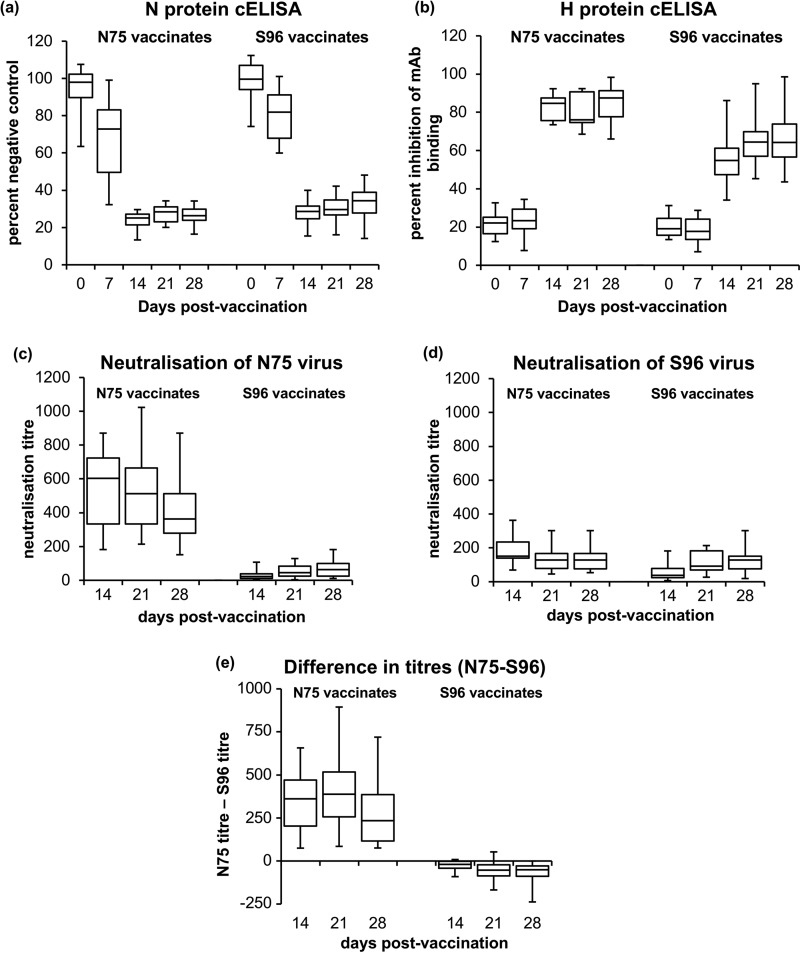
Antibody response in goats to PPRV vaccines. Goats were vaccinated with N75 or S96, and serum samples were taken at 0, 7, 14, 21, and 28 dpv. Sera taken at all time points were assayed for antibodies to the PPRV N protein (a) and the PPRV H protein (b) using available cELISA kits. Sera taken at 14, 21, and 28 dpv were additionally assayed for neutralization of PPRV; neutralizing titers were determined against N75 vaccine virus (c) and S96 vaccine virus (d). (e) The differences in titers (titer against N75 virus – titer against S96 virus) for each individual serum was calculated, and these differences are plotted. Note that the manufacturer’s recommended calculation for the N protein cELISA kit (in panel a) gives values that decrease as the amount of anti-N antibody increases (percentage of negative control), whereas that for the H protein cELISA kit (in panel b) gives values that increase as the amount of competing antibody increases (percent inhibition of binding of MAb). The data are presented as box-and-whisker plots, in which the bars span the minimum and maximum values, and the box shows the range from the first to the third quartile. The central horizontal line in each box shows the median value. The numbers of samples assayed at each time point shown were as follows: 25/19/25/19/24 (a), 15/10/15/10/14 (b), and 15/15/15 (c to e).

The most commonly used way to assess the antibody responses to vaccination with live PPRV vaccines, is to use the commercially available competition enzyme-linked immunosorbent assay (cELISA) kits, which measure antibodies directed to the viral nucleocapsid (N) protein (22) or the viral surface attachment (H) protein (23). For both of these assays, the viral antigen used to coat the ELISA plates is based on the N75 vaccine. As can be seen in Fig. 2, vaccination with either N75 or S96 gave rise to antibodies that reacted strongly in either cELISA. Animals vaccinated with N75 had slightly stronger responses (showed greater competition) in the N protein cELISA (Fig. 2a), statistically significant at 7, 14, 21, and 28 dpv (*P* = 0.006, 0.003, 0.01, and 0.0004, respectively). The antibody response as measured in the H protein cELISA appeared to develop slightly more slowly (Fig. 2b), with no significant detection of anti-H antibodies in sera from either group of vaccinates at 7 dpv. However, thereafter both groups showed clear increases in anti-H antibodies, with sera from N75 vaccinates again showing greater competition than sera from S96 vaccinates (*P* ≤ 0.0001 for this comparison at 14, 21, and 28 dpv).

An alternative way of quantifying development of anti-virus antibody is to determine the ability of sera to prevent virus infection of susceptible cells, expressed as the virus neutralization titer. As this assay is more time-consuming, it was only performed for three sets (i.e., 15 animals for each group of vaccinates). An advantage of this assay is that, not being confined to a specific kit with antigen derived from only N75, all sera could be tested for the ability to neutralize both N75 and S96 viruses, i.e., both homologous and heterologous neutralization. Since it was reasonable to assume a zero titer for unvaccinated animals in the United Kingdom (and a selection of day 0 sera indeed showed no effect on virus infection), and since the cELISA data suggested that the neutralizing titer at 7 dpv would also be very low, we assayed the neutralization titer in samples taken at 14, 21, and 28 dpv.

These studies showed that N75 vaccinates had a higher titer against N75 virus than did the S96 vaccinates at all the time points assayed (Fig. 2c) (*P* < 0.0001). Interestingly, this group of vaccinates also had a higher titer against S96 virus at 14 dpv (*P* < 0.0001), though this difference disappeared by 21 dpv (Fig. 2d). In general, the N75 vaccinates seemed to have reached their maximum titer against either target virus by 14 dpv, while the neutralizing titers in the sera from the S96 vaccinates continued to increase up to 28 dpv. We also looked at the difference in titers of the individual sera against N75 and S96 (Fig. 2e), as opposed to the differences between the two populations of titers; we found that the N75 vaccinates had a significantly higher titer against their homologous target at all time points measured (*P* ≤ 0.0001), while S96 vaccinates have a higher titer against their homologous target at 21 and 28 dpv (*P* = 0.018 and *P* = 0.032, respectively). The neutralization data therefore agree with the cELISA data that N75 vaccinates have a stronger antibody response (higher concentration and/or higher affinity of anti-PPRV antibodies) when measured against the homologous protein or virus and show that the sera from these animals also have, at least initially, a stronger antibody response even against a heterologous target. S96 vaccinates have a slower-developing but less target-specific antibody response, with only a small difference between their recognition of homologous and heterologous targets.

### T cell response to vaccination.

Several approaches were tried to measure the development of PPRV-specific T cells after vaccination. Pools of peptides covering the PPRV H protein sequence (24) did not give any detectable T cell response in peripheral blood mononuclear cells (PBMCs) from vaccinated goats (data not shown). In addition, nonradioactive assays of cell proliferation (“cell trace”) showed no detectable specific proliferation in response to peptides or total virus protein. We were able to detect PPRV-specific T cell proliferative responses in PBMCs from vaccinated animals using incorporation of [^3^H]thymidine after stimulation with either crude virus antigen or heat-inactivated virus (HI-virus), of which the former gave more pronounced PPRV-specific cell proliferation. Using this technique, the cell-mediated immune responses were studied in each group of vaccinated animals using both N75 and S96 virus proteins, i.e., both homologous and heterologous targets.

**FIG 3:**
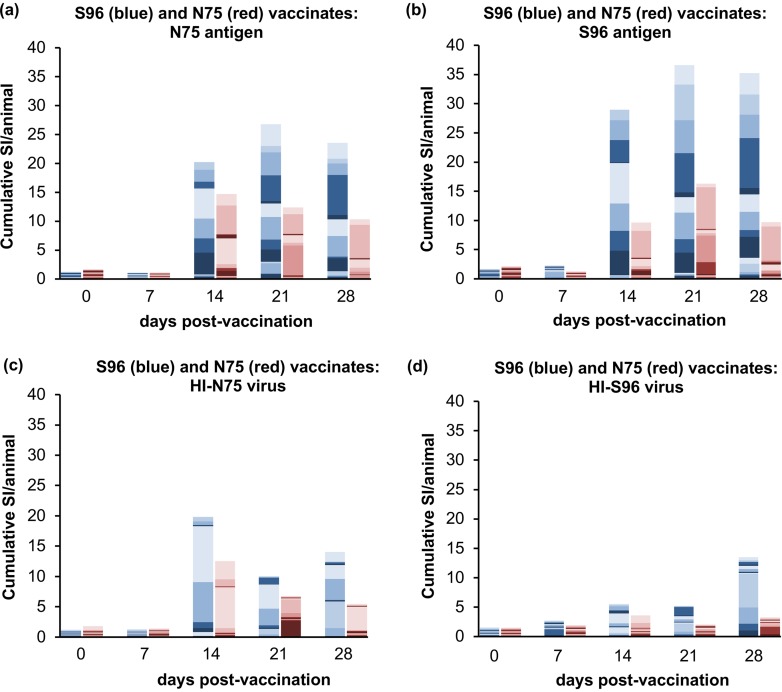
T cell proliferative responses in goats after PPRV vaccination. Goats were vaccinated with N75 or S96 vaccine, and PBMCs were prepared from heparinized blood at 0, 7, 14, 21, and 28 dpv. Proliferative responses were measured by incorporation of [^3^H]thymidine as described in Materials and Methods. Proliferation was stimulated with protein from cells infected with N75 (a), protein from cells infected with S96 virus (b), heat-inactivated preparations of N75 virus (c), or heat-inactivated preparations of S96 virus (d). The stimulation index (SI) was measured as the proliferation relative to that seen in PBMCs incubated with protein from uninfected cells (a and b) or mock viral preparations from uninfected cells (c and d). The data are presented as a stacked bar plot showing the SI for each animal, using different shades to delineate the contribution of different animals to the cumulative SI for the group of vaccinates at that time point. Data from animals vaccinated with S96 (*n* = 15) are in blue shades; data from animals vaccinated with N75 (*n* = 14) are in red shades. To allow for the differing number of animals in each group, the values have been scaled before plotting by dividing by the number of animals in the group.

Both groups of vaccinates showed an increased stimulated proliferation of PBMCs from 14 dpv, and the proliferation within each group was approximately the same whether stimulation was carried out with homologous or heterologous antigen (Fig. 3a and b). A similar pattern was found using HI-virus as the stimulating antigen (Fig. 3c and d), although the proliferation observed was in general less. The stimulation of PBMCs from S96 vaccinates was clearly significantly increased over 0 dpv at 14, 21 and 28 dpv, whether the stimulus was homologous (S96 antigen) or heterologous (N75 antigen) (*P* ≤ 0.0003 for all comparisons). The response in N75 vaccinates was smaller and, perhaps due to the high level of background in samples taken prevaccination in this group of animals, not significantly different from 0 dpv at any subsequent time. However, compared to 7 dpv, the PPRV-specific responses in the vaccinated animals were significantly increased at 14, 21, and 28 dpv for homologous (N75) antigen (*P* = 0.004, *P* = 0.017, and *P* = 0.016, respectively) and at 21 and 28 dpv for the heterologous antigen (*P* = 0.027 and *P* = 0.017, respectively).

Comparing the two groups of vaccinates, the data in Fig. 3 suggested that S96 vaccinates had a greater T cell proliferative response than the N75 vaccinates, regardless of the stimulating antigen; however, this only reached statistical significance at 28 dpv (*P* = 0.0005 and *P* = 0.045 for S96 and N75 antigens, respectively).

**FIG 4:**
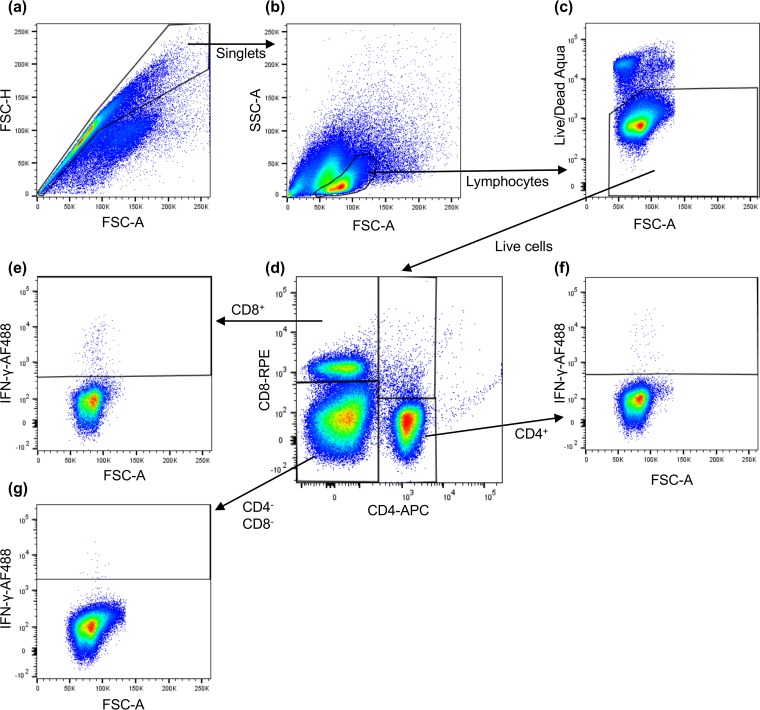
Gating strategy for identifying PPRV-specific CD4^+^ and CD8^+^ T cells. PBMCs were stimulated with live virus or antigen for 18 h and then for an additional 6 h in the presence of the Golgi transport inhibitor Golgi-Plug. The cells were then labeled with anti-CD4 and anti-CD8 MAbs and Live/Dead stain, after which they were fixed/permeabilized and labeled with anti-IFN-γ. The numbers of labeled cells were determined by flow cytometry using a MACSQuant, and the data were analyzed with FlowJo X. An example data set is shown for PBMCs taken from an animal vaccinated with S96 at 14 dpv and stimulated with S96 virus. The cells were gated successively for singlets (a), lymphocytes (b), live cells (c), and then by the presence or absence of the surface markers CD8 and CD4 (d). The percentages of IFN-γ^+^ cells was obtained for CD8^+^ cells (e), CD4^+^ cells (f), and CD4^–^ CD8^–^ cells (g) are depicted.

These data showed that both vaccines induce some level of T cell-mediated response, but do not give any information as to which cell types are being induced. We therefore combined the labeling of lineage-specific T cell membrane proteins with intracellular cytokine staining (ICS) of gamma interferon (IFN-γ) (25) and used flow cytometry to identify the fractions of CD4^+^, CD8^+^, and CD4^–^ CD8^–^ cells that were responding to PPRV antigens by increased production of IFN-γ. The gating strategy used to define the three populations of cells is shown in Fig. 4. For these studies, the best responses were obtained with live PPRV virus as the stimulating antigen, although the virus antigen preparation was also effective at stimulating PPRV-specific CD4^+^ cells. In all cases, the specific response was taken as the fraction of IFN-γ^+^ cells in each group after subtracting the fraction of such cells seen after stimulation with a mock virus or antigen preparation.

**FIG 5:**
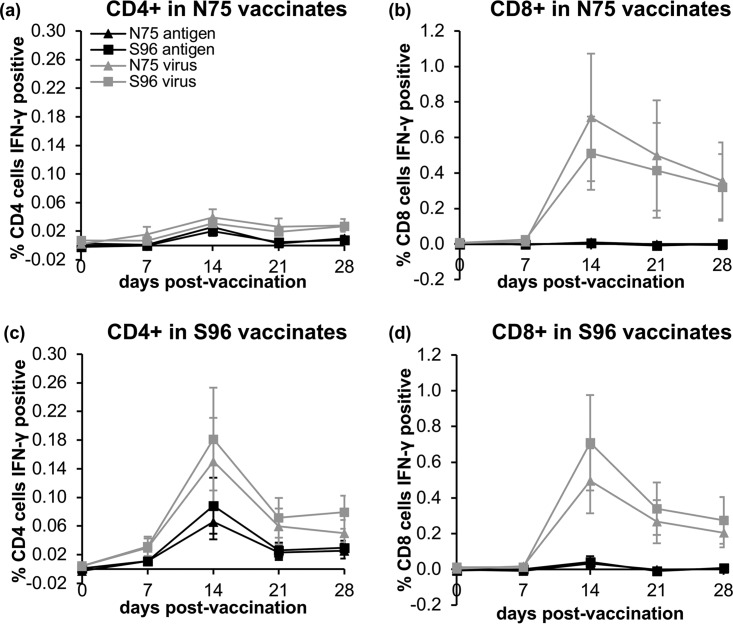
PPRV-specific IFN-γ-producing CD4^+^ and CD8^+^ T cells in goats following PPRV vaccination. Goats were vaccinated with N75 or S96 vaccine and PBMCs were prepared from heparinized blood at 0, 7, 14, 21, and 28 dpv. T cell subtypes responding to live PPRV virus or inactivated PPRV antigen by producing IFN-γ were determined in triplicate by intracellular labeling of IFN-γ and flow cytometry as described in Materials and Methods. The gating strategy for identifying IFN-γ^+^ CD8^+^ or CD4^+^ T cells was as shown in Fig. 4. The percentages of total CD4^+^ T cells (a and c) or CD8^+^ T cells (b and d) that responded to PPRV were calculated by subtracting the percentages observed after stimulation with mock antigen or virus preparations. The graphs show the mean specific percentages of IFN-γ-producing cells observed in response to the indicated stimulation of PBMCs from five N75 (a and b) and five S96 (c and d) vaccinates. Error bars show the standard errors of the mean.

Both groups of vaccinates showed a clear development of PPRV-specific CD8^+^ cells (Fig. 5); analysis of variance showed no difference between vaccines, but a clear effect of dpv, although the stimulation was only statistically significant at 14 dpv (*P* = 0.006). The CD8^+^ cells were not activated by incubation with the virus antigen preparation. The S96 vaccine also elicited PPRV-specific CD4^+^ T cells, with the numbers of reacting CD4^+^ cells in S96 vaccinates significantly increased at 14, 21 and 28 dpv (*P* ≤ 0.02 for all cases), and the same effect was seen for stimulation with either live virus or virus antigen. In cells from N75 vaccinates, there appeared to be a slight increase in the percentage of PPRV-specific CD4^+^ T cells, but the change did not reach statistical significance. No change was seen in the numbers of IFN-γ^+^ CD4^–^ CD8^–^ cells at any time (data not shown).

### Protection against challenge.

**FIG 6:**
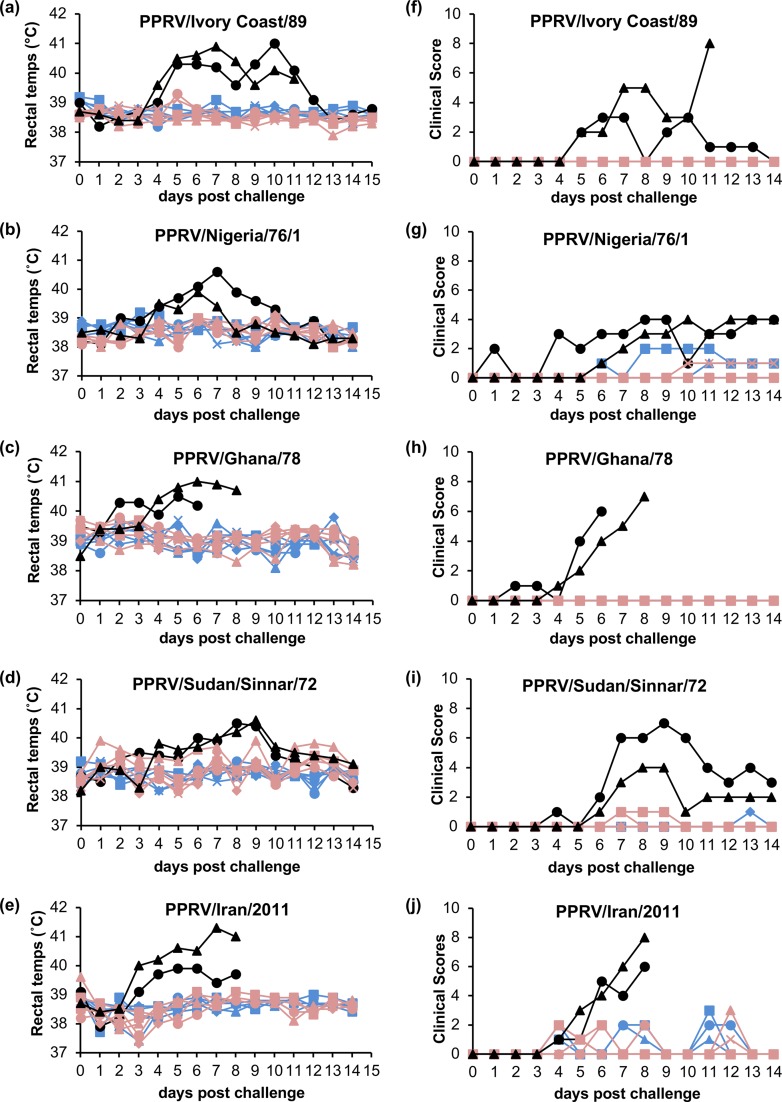
Rectal temperatures and clinical scores in experimental animals after challenge with wild-type viruses. The rectal temperatures (a to e) and clinical scores (f to j) of each experimental animal were recorded daily after challenge with the indicated wild-type virus. Data from individual unvaccinated animals are shown in black, data from S96 vaccinates are shown in blue, and data from N75 vaccinates are shown in red.

Groups of animals were challenged with one of five wild-type viruses: PPRV/Ivory Coast/89 (lineage I), PPRV/Nigeria/76/1 (lineage II), PPRV/Ghana/78 (lineage II), PPRV/Sudan/Sinnar/72 (lineage III), and PPRV/Iran/2011 (lineage IV). In each case, two unvaccinated goats were exposed to the challenge virus at the same time, to act as controls. The isolates used exhibited, in UK goats, a range of virulence, from very mild (Nigeria/76/1), to moderate (Sudan/Sennar/72), to severe (Ivory Coast/89, Ghana/78, and Iran/2011). In the case of the viruses causing severe disease, one or both of these unvaccinated control animals had to be euthanized before the termination of the experiment, as soon as the humane endpoint of this moderate protocol was reached. Rectal temperatures and clinical score profiles for these animals are given in Fig. 6.

None of the vaccinated animals showed any significant clinical signs at any stage; one or two animals in either group of vaccinates occasionally showed a slight congestion in the conjunctiva, or a transient cough, but this appeared to be an artifact of the closed environment of isolation units and unrelated to the study protocol, as we have observed this also in untreated animals. Notably, none of the vaccinated animals showed any detectable pyrexia (Fig. 6a to e). No difference was seen in the temperature profiles or clinical scores of the two groups of vaccinates in any of the five challenge studies, although the unvaccinated control animals had clinical scores and temperature profiles that were significantly different from the vaccinates (*P* < 0.05) in each case.

**FIG 7:**
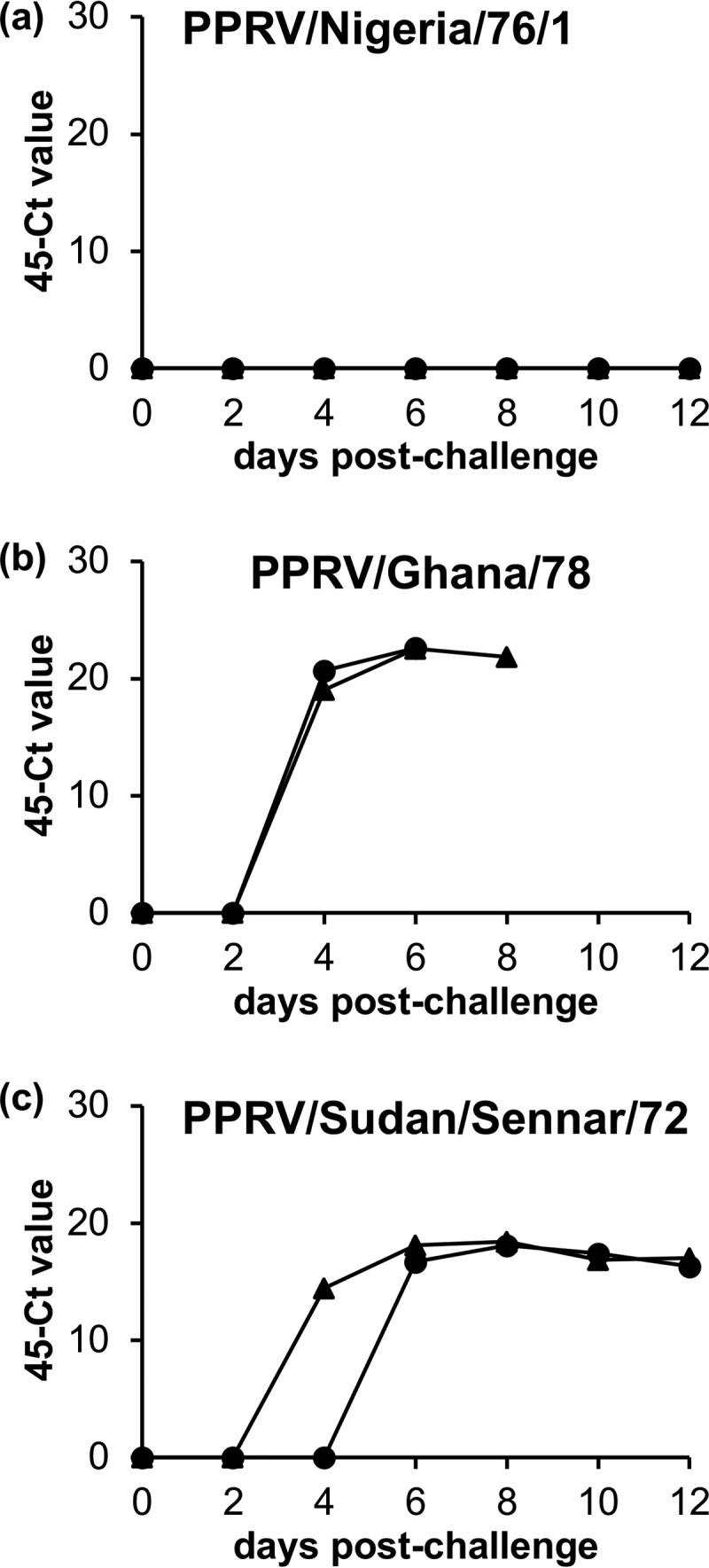
Detection of PPRV genome by RT-qPCR in unvaccinated controls after challenge. EDTA-blood was collected every second day after challenge, and 1-ml aliquots were frozen for later analysis. PPRV RNA was detected using the one-tube RT-qPCR technique (see Materials and Methods). Points are the means of duplicate PCRs, results are displayed as “45 – *C_T_*” values. Graphs depict the results for mock-vaccinated controls infected with PPRV/Nigeria/76/1 (a), PPRV/Ghana/78 (b), or PPRV/Sudan/Sennar/72 (c). None of the vaccinated animals tested had detectable PPRV RNA in any blood sample taken postchallenge (data not shown).

We also carried out reverse transcription real-time PCR (RT-qPCR) for PPRV RNA in blood samples from animals in three of the vaccination/challenge studies (appropriate samples were not collected for the PPRV/Ivory Coast/89 and PPRV/Iran/2011 challenges). These challenges represent mild, moderate, and severe disease. PPRV RNA was not detected by this technique in samples from any group of vaccinates exposed to challenge with any of these three PPRV isolates (not shown), although PPRV RNA was clearly detected from 4 to 6 days postchallenge (dpc) onward in samples from unvaccinated animals infected with PPRV/Ghana/78 or PPRV/Sudan/Sennar/72 (Fig. 7b and c). Samples from unvaccinated controls infected with PPRV/Nigeria/76/1 were determined to be negative by RT-qPCR at all time points postchallenge (Fig. 7a).

### Immune response following challenge with wild-type PPRV.

**FIG 8:**
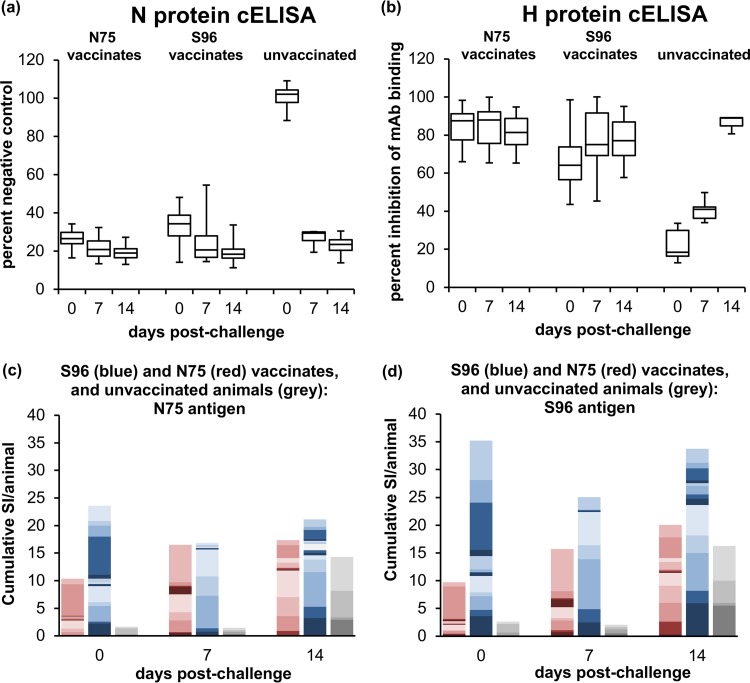
Immune responses in goats after challenge with PPRV. Goats vaccinated with live PPRV vaccines were challenged 28 dpv, along with unvaccinated controls. Heparinized blood and serum was collected from each animal at 0, 7, and 14 days postinfection (dpi). (a and b) Serum samples were assayed for antibodies to the PPRV N protein (a) and the PPRV H protein (b) using available cELISA kits. The N protein cELISA was applied to all samples from all five challenge studies; the H cELISA was only applied to samples from animals challenged with PPRV/Ivory Coast/89, PPRV/Nigeria/76/1, and PPRV/Iran/2011. The numbers of serum samples assayed at the time points shown were as follows: 25/25/20 for vaccinates and 8/7/4 for controls (a) and 14 at each time point for N75, 15 at each time point for S96 vaccinates, and 6/6/3 for controls (b). The data in panels a and b are presented as box-and-whisker plots, in which the bars span the minimum and maximum values and the boxes shows the ranges from the first to the third quartile. The central horizontal line in each box shows the median value. (c and d) PBMCs were prepared from the heparinized blood, and the proliferation of cells in response to PPRV antigen was assayed as described for Fig. 3. Proliferation was stimulated with protein from cells infected with N75 (c) or protein from cells infected with S96 virus (d). The SI was measured as the proliferation relative to that seen in PBMCs incubated with protein from uninfected cells. The data are presented as stacked bar plots showing the SI for each animal, using different shades to delineate the contributions of different animals to the cumulative SI for the group of vaccinates at that time point. Data from individual animals vaccinated with N75 are in red shades, data from individual animals vaccinated with S96 are in blue shades, and data from individual unvaccinated animals are in gray shades. To allow for the various number of animals in each group, the values have been scaled before plotting by dividing by the number of animals in each group. Data were obtained for samples from animals challenged with PPRV/Nigeria/76/1 and PPRV/Sudan/Sennar/72; data were also obtained for the vaccinates challenged with PPRV/Iran/2011, but not from unvaccinated animals infected with this virus, whose white cell counts at 7 dpi were too low for the assay to be carried out and were euthanized before 14 dpi. The numbers of PBMC samples assayed at each time were 14/9/12 for N75 vaccinates, 15/10/14 for S96 vaccinates, and 4/4/4 for unvaccinated animals. Note that the data for 0 dpc are the same as that shown in Fig. 2 and 3 for 28 dpv and are included here for comparison.

Antibody and T cell responses were assessed in vaccinates and control animals during the 14 days after infection with challenge virus. The unvaccinated control animals, as expected, developed a strong anti-N antibody response at 7 dpc (*P* < 0.0001) and which did not increase significantly between 7 and 14 dpc (Fig. 8a). Levels of anti-N antibody also increased in both groups of vaccinates at 7 dpc (*P* ≤ 0.005), and also showed no significant increase between 7 and 14 dpc (Fig. 8a). Control animals also developed a strong anti-H response (Fig. 8b); as observed for the primary antibody response to the vaccines, the anti-H response developed slightly more slowly, so the level of competing antibodies was significantly increased at 7 dpc (*P* = 0.0013) and further increased at 14 dpc (*P* < 0.0001). The levels of anti-H antibody were also boosted in S96 vaccinates, showing a significant increase at 7 dpc (*P* = 0.001) but no further increase at 14 dpc. In contrast, the N75 vaccinates showed no significant change in anti-H antibody levels, perhaps because these were close to the maximum detectable by this assay before challenge.

T cell proliferation assays (Fig. 8c and d) showed that the surviving control animals developed a PPRV-specific response by 14 dpc (*P* = 0.0062), although samples could only be obtained from the control animals infected with PPRV/Nigeria/76/1 and PPRV/Sudan/Sennar/72, as the animals infected with PPRV/Iran/2011 had too few PBMCs at 7 dpc, and had been euthanized before 14 dpc. The proliferation assay was established after the completion of the experiments involving challenge with PPRV/Ivory Coast/89 and was not carried out for the animals in the PPRV/Ghana/78 challenge study due to time and sample restrictions. The N75 vaccinates showed a boost (*P* = 0.0075) in proliferative responses to both viral antigens after challenge. The S96 vaccinates, on the other hand, showed no significant change in cell proliferation following challenge, which may reflect that the response seen in cells from these animals was already, prior to challenge, at the level reached by cells from the other groups after infection/boost (Fig. 8c and d). There was no significant difference between the proliferative responses seen in any of the three groups of vaccinated/unvaccinated animals at 14 dpc.

## DISCUSSION

We have shown that both N75 and S96 vaccine strains of PPRV gave complete clinical protection against challenge with field isolates representing all four genetic lineages of PPRV. Vaccinated animals demonstrated only occasional minor clinical signs such as slight congestion of ocular membranes, without discharge. Reddened ocular membranes occurred inconsistently both before and after challenge and might have been enhanced by the closed environment within the isolation facilities. In addition, as the goats in this study were of the white Saanen breed with a natural pallor, it could be that changes in membrane color were more obvious than they would be in other breeds. In the light of these observations, this clinical sign on its own is probably not as useful a marker of PPRV infection in this breed as nasal discharge and elevated rectal temperatures.

Where tested, neither group of vaccinates had detectable PPRV RNA in their blood, even when challenged with highly virulent PPRV/Ghana/78, infection with which gave rise to high levels of viral RNA in circulating blood in unvaccinated animals, as determined by RT-qPCR, providing further evidence of the general cross-protection provided by both vaccines. An important additional observation from these studies was that a very mild strain of PPRV, the Nigeria/76/1 isolate, had no detectable viral RNA in blood samples from unvaccinated challenge control animals. This means that an animal infected with such a virus may not be identified as PPRV-infected by routine testing. Very mild rinderpest virus isolates were known to circulate during the later stages of the rinderpest eradication campaign (e.g., see references 26, 27, and 28), and sometimes went undetected because of their low pathogenicity. It will be important to determine whether similar PPRV strains are transmitted and may therefore be circulating undetected in some populations.

In terms of the specific immune responses following vaccination, both vaccines induced both antibody and T cell-mediated responses. While the N75 vaccine appeared to be superior at eliciting neutralizing antibodies (particularly against its homologous virus), the S96 vaccine seemed to be better at inducing T cell responses, particularly PPRV-specific CD4^+^ cells, as seen in the IFN-γ assay and implied by the proliferation assay data. The virus neutralization tests gave a more balanced picture than the cELISAs, since they give an indication of the levels of neutralizing (and therefore presumably protective) antibodies present in serum, while the cELISAs measure the presence of a particular subset of anti-PPRV antibodies which, in the case of anti-N protein antibodies, are unlikely to be protective, since anti-N antibodies are not neutralizing. It is clear from the studies here that vaccination with N75 gave rise to high-titer antibodies directed against PPRV/Nigeria/75/1-specific neutralizing epitopes, since there was relatively little difference in the titers of the two groups of sera against S96, but a very large difference in titers against N75. This suggests that both vaccines elicit good titers of antibodies that are generally cross-protective, but there is an additional strong epitope on the N75 vaccine strain. It would be interesting to determine whether this epitope is specific to the Vero cell-adapted vaccine or is common to other lineage II viruses.

Several antigens were assessed for their ability to stimulate the production of IFN-γ for the ICS assay, but by far the most successful stimulant for PPRV-specific IFN-γ production was live PPRV. Both vaccines induced CD8^+^ T cells that produced IFN-γ in response to live virus stimulation. These data suggest efficient processing of antigen into the major histocompatibility complex class I presentation pathway, which in turn suggests that PPRV is infecting a subset of cells within the PBMC population, presumably cells expressing the PPRV receptor SLAM/CD150. It would be interesting to explore whether this is indeed the case and which cells are infected. Morbilliviruses, particularly the attenuated vaccine strains, infect PBMCs relatively poorly, unless the expression of canine signaling lymphocyte activation marker (SLAM) is first stimulated by incubation with mitogens such as concanavalin A (29–32). While both vaccines elicited similar numbers of PPRV-specific CD8^+^ T cells, it would appear from the data presented here that the S96 vaccine is better than the N75 vaccine at eliciting PPRV-specific CD4^+^ T cells. These CD4^+^ cells are most likely the underlying reason for the difference in the proliferation assay data between S96 and N75 vaccinates, where cells from the former proliferated significantly more strongly than cells from the latter. This difference between the detailed immune response to the two vaccines, of course, may be dependent on the genotype of the hosts, since differences in genotype are known to affect the levels of specific types of immune responses in measles virus vaccination (e.g., see references 33, 34, and 35). However, it is of note that these differences in the details of the response did not affect the protection from disease. In our studies, neither the proliferation assay nor the ICS assay showed any significant difference in the responses to heterologous or homologous antigens, suggesting the dominant epitopes are common to at least both lineage II and lineage IV. The presence of strong epitopes common to all lineages provides a mechanism for the broad cross-protection seen between PPRV strains and indeed between PPRV and rinderpest virus (21, 36).

One of the primary arguments for using lineage-specific vaccines is the fear that a vaccine could revert to virulence and lead to multiple genetic lineages of PPRV circulating in the field (13). This seems an unfounded concern as the live attenuated vaccines have been tested and used for decades in multiple countries with no adverse effects, including no adverse events in pregnant animals (13, 37, 38). Indeed, a greater risk arises from the introduction of new vaccines that may not have been so well characterized. A more realistic concern is the creation of hybrid lineage viruses through recombination between cocirculating viruses. Studies using computer analysis of genome sequences have suggested that recombination occurs between homologous paramyxoviruses (see reference 39 and references therein). Such recombination may eventually be shown also for PPRV, if an animal is infected with two significantly different wild-type viruses. The studies reported here demonstrate that there are conserved protective epitopes common to all lineages of the virus and which will therefore also be present on any interlineage recombinant, meaning any such recombinant will also be susceptible to the immune protection provided by either of these vaccines.

While the N75 and S96 vaccine strains are effective, they are derived from viruses isolated 43 and 22 years ago, respectively, and there may be significant genetic drift between these strains and those circulating now. In the case of Newcastle disease virus, which is also a paramyxovirus and causes a widespread and economically important diseases in chickens, turkeys, and other birds, the established live attenuated virus vaccines are also now many decades old. While these vaccines are effective at preventing disease caused by modern strains of the virus when administered correctly (40–43), they are not as effective at preventing virus shedding as vaccines that are genotypically matched to the wild-type challenge virus (42–47). Shedding is particularly important in the case of this avian disease, since many wild species of birds are also susceptible and can transmit the virus. The situation may be different in the case of a disease affecting sheep and goats, which are not as intensively housed, and where there does not appear to be a wildlife reservoir. It has been shown that N75 vaccine does not completely prevent shedding, as represented by detectable viral RNA in nasal, ocular and rectal swabs, from challenged animals (48); however, that study did not measure shedding of infectious virus, and the fact that this lineage II vaccine has been successfully used in both China and Morocco to eradicate disease caused by lineage IV viruses suggests that, at the moment, the existing PPRV vaccines are sufficiently cross-protective to prevent transmission in vaccinated animals.

We have shown here that two vaccine strains, one originating in Nigeria and the other originating in India, are each capable of protecting goats against infection with field strains of any genetic lineage, including a recent isolate, confirming that any properly characterized live attenuated PPRV vaccine that is available can be utilized to protect livestock, irrespective of the local circulating strains. It is clear, however, that it will be important for those involved in the current efforts to control and eventually eradicate PPR to continue to monitor the effectiveness of the vaccines in use, and better information on the shedding of infectious virus (as opposed to virus antigen or RNA) by vaccinated animals would be useful.

## MATERIALS AND METHODS

### Viruses, including vaccines.

All virus stocks were cultured in Vero cells expressing canine signaling lymphocyte activation marker (SLAM), the morbillivirus receptor (Vero-Dog-SLAM [VDS]) (49). The vaccine viruses used were recombinant PPRV/Nigeria/75/1 (50) and PPRV/India/Sungri/96 vaccine (14), the original stock of which was the gift of MSD Animal Health. In order to eliminate any effect of differences in the way the two vaccines were prepared, the vaccine stocks were grown and titers determined in our laboratory, using the same cell stocks, media, and processing. They were passaged one to two times from rescue (N75) or from the original commercial stock (S96). For serum neutralization tests we used the same viruses. The field isolates used and their passage histories since last isolated from an animal were PPRV/Nigeria/76/1 (BK6/V3/VDS1), PPRV/Sudan/Sennar/72 (BK1/LK1/V2/VDS1), PPRV/Ivory Coast/89 (VDS1), and PPRV/Ghana/78 (LK1/V1/VDS1), all of which were from the Pirbright Institute virus archive, and PPRV/Iran/2011 (originally called PPRV/Kurdistan/2011 [51]) (CV1-S1/VDS2), a gift from Bernd Hoffman, FLI, Germany. Virus stocks were grown, and titers were determined on VDS as previously described (52).

### Vaccination/challenge protocol.

All animal studies were carried out under licenses PPL 70/7199 and PPL70/8833 issued by the Home Office of the United Kingdom in accordance with relevant legislation and after approval by the Pirbright Institute Animal Welfare and Ethical Review Board. Outbred goats were sourced from commercial farms. Animals entered the secure isolation units 7 days before the start of the procedure to acclimatize to the new husbandry regime and were provided with daily enrichment during the trials.

The core study, in which animals were vaccinated using live PPRV vaccines, and then challenged with a wild-type virus, was repeated five times, once for each wild-type virus. In each core study, two groups of goats (9 to 12 months old), each consisting of five animals, were inoculated with a standard dose (2 × 10^4^ 50% tissue culture infectious doses [TCID_50_] determined in VDS cells [VDS TCID_50_], equivalent to 10^3^ TCID_50_ determined in Vero cells) of S96 or N75, given subcutaneously. Two cohoused animals were left unvaccinated to act as controls for the challenge virus. After 4 weeks, all the animals were infected with 2 × 10^5^ VDS TCID_50_ of challenge virus. Blood samples were collected on various days postvaccination (dpv) and days postchallenge (dpc) for different assays. In the first vaccination/challenge study carried out (PPRV/Ivory Coast/89) this challenge was given intranasally; however, there was concern that it was more difficult to exactly control the dose of challenge virus administered to each animal using this route. To ensure uniformity of the challenge in each animal, and since our previous studies had not identified any difference in the disease elicited by virus administration intranasally or by injection (52), the remainder of the challenges were given subcutaneously.

Clinical scores for PPR disease were calculated based on rectal temperatures and other clinical signs. A score of 1 was given for rectal temperatures 0.1 to 2°C above normal for the animal, and a score of 2 was given for temperatures >2°C above normal. Similarly, a score of 1 was given for ocular or nasal congestion, congestion of the gums, a soft stool, a cough, or apathetic behavior; a score of 2 was given for visible ocular or nasal discharge, one to two lesions in the gums, watery diarrhea, or reluctance to walk or stand; and a score of 3 was given for necrotic oral lesions. The cumulative score on each day was recorded as the final clinical score. Animals reaching clearly defined humane endpoints of a moderate severity were euthanized humanely.

### Assays for anti-PPRV antibodies.

Serum samples were obtained from goats by venipuncture into plain vacutainers. After clotting, the tubes were centrifuged at 2,000 × *g* for 20 min; the serum removed and either stored at –80°C or heat treated at 56°C for 2 h (inactivating both complement and any remaining virus) and then stored at –20°C. Antibodies specific for PPRV proteins were measured using the N protein-specific cELISA kit available from ID Vet (Garbles, France), or the H protein-specific cELISA was developed at the Pirbright Institute (23). Sera were assayed in duplicate (N protein cELISA) or triplicate (H protein cELISA), and results were calculated in accordance with the manufacturers’ instructions. Statistical analysis was based on the mean of technical replicates. Neutralizing titers against N75 and S96 were determined in VDS cells as previously described (21). The titer was taken to be the reciprocal of the dilution at which 50% of wells showed no cytopathic effect and was calculated using the Spearman-Kärber method (53).

### Preparation of PBMCs.

PBMCs were prepared from heparinized goat blood obtained by venipuncture. Approximately 30 ml of heparinized blood was mixed with 20 ml Dulbecco phosphate-buffered saline (PBS; Thermo Fisher) and then layered over 15 ml of Ficoll-Paque Plus (GE Healthcare) in Leucosep tubes prepared according to the manufacturer’s instructions (Greiner). These gradients were centrifuged at 800 × *g* for 20 min at 20°C with no brake. Mononuclear cells above the polyethylene barrier were collected and then washed in Hanks balanced salt solution (HBSS), resuspended in 10 ml of 1× RBC lysis buffer (Santa Cruz), and incubated at room temperature for 5 min. After centrifugation at 330 × *g* for 5 min, the cells were washed once more in 10 ml of HBSS and resuspended in 10 ml of RPMI 1640 (Thermo Fisher) supplemented with 5% (vol/vol) fetal bovine serum (FBS), 1× MEM nonessential amino acid Solution (Thermo Fisher), 1 mM sodium pyruvate (Thermo Fisher), 0.5 μg/ml gentamicin (Sigma), and 50 μM 2-mercaptoethanol (Sigma) (proliferation medium). Viable PBMC counts were determined by Trypan Blue exclusion using a Nexelom T4 automated cell counter, and the cells were either used immediately or stored frozen over liquid nitrogen in 90% FBS–10% dimethyl sulfoxide until used. For use, cryopreserved cells were thawed quickly in a 37°C water bath, diluted slowly with 4 volumes of proliferation medium prewarmed to 37˚C, and then rapidly diluted with a further 4 volumes of medium. The cells were pelleted at 330 × *g* for 10 min, washed with a further 10 ml of proliferation medium, and finally resuspended in proliferation medium before the viable cells were counted. We did not observe any consistent difference between the results of assays with frozen and fresh cells. Fresh cells were used for all flow cytometric analyses of T cell IFN-γ responses; fresh cells were used for T cell proliferation studies on all samples from the set of vaccinates challenged with PPRV/Sudan/Sennar/72 and on almost all samples from the set of vaccinates challenged with PPRV/Nigeria/76/1, with the exception of samples from 7 dpc; T cell proliferation studies on samples from the set of vaccinates challenged with PPRV/Iran/2011 were all performed using cryopreserved cells.

### Preparation of labeled antibodies.

Mouse monoclonal antibodies (MAbs) were conjugated to AlexaFluor488 using a specific antibody labeling kit (Thermo Fisher), whereas MAb conjugation with other fluorochromes (allophycocyanin [APC] and R-phycoerythrin [RPE]) was carried out using Lightning Link kits (Innova Biosciences); in all cases the manufacturers’ instructions were followed directly. Conjugated antibodies were titrated on goat PBMCs before use to determine the optimal working concentration and stored at 4°C. The antibodies used included anti-CD8 (clone CC58 [54]), anti-CD4 (clone 17D [ECACC 91060551] [55]), and the isotype control antibodies TRT1 and TRT3 (56).

### Antigen preparations for T cell response assays.

To generate crude virus antigen preparations, flasks (10 by 175 cm^2^) of VDS were infected with either PPRV Nigeria/75/1 or PPRV Sungri/96 at a multiplicity of infection of 0.1, followed by incubation at 37°C and 5% CO_2_ until the cytopathic effect was extensive (usually 2 to 3 days). The same numbers of uninfected cells were processed identically to produce negative-control VDS antigen. Preparation of virus antigens from the infected and control cell pellets was carried out as previously described (21). Protein concentration was determined by Coomassie (Bradford) protein assay kit (Thermo Fisher). Antigen was diluted to 1 mg/ml in 10% sucrose–PBS, and aliquots of this antigen were stored at –20°C.

Preparations of virus or HI-virus for use in T cell response assays were tissue culture supernatants from similarly infected or noninfected VDS, used either directly or following heat inactivation at 56°C for 2 h.

### T cell proliferation assay.

Thymidine incorporation assays were carried out essentially as previously described (21), except that some assays were performed on cryopreserved cells. PBMCs (2 × 10^5^) were plated in 96-well U bottomed plates and exposed in quadruplicate wells to each of the following PPRV and control antigen preparations: medium only, 5 μg/ml concanavalin A (Sigma-Aldrich), 20 μg/ml VDS cell control antigen, 20 μg/ml N75 antigen, 20 μg/ml S96 antigen, and a 1:2 dilution of HI-N75, HI-S96, or a HI-VDS cell mock virus preparation. Cells were incubated at 37˚C, 5% CO_2_ for 5 days; 0.037 MBq [^3^H]thymidine (Perkin-Elmer) in 30 μl of RPMI 1640 was then added per well, and the cells were incubated overnight at 37°C and 5% CO_2_. Cells were harvested onto prewet glass fiber filter mats (Perkin-Elmer) using an automated harvester. The filter mats were dried before being sealed in sample bags with 3.5 ml of BetaScint scintillation liquid (Perkin-Elmer). Scintillation counts were recorded using a 1450 MicroBeta TriLux (Wallac) and MicroBeta Windows Workstation V4.70.05 software. Stimulation indices (SI) were calculated by dividing the mean counts per minute (cpm) for cells incubated with specific antigen by the mean cpm for cells incubated with the mock antigen.

### Flow cytometric analysis of T cell interferon gamma responses.

PBMCs (10^6^) were plated in 96-well U-bottom plates and incubated in triplicate wells with each of the following PPRV and control preparations: medium only, 5 μg/ml pokeweed mitogen (Sigma-Aldrich), 20 μg/ml VDS cell control antigen, 20 μg/ml N75 antigen, 20 μg/ml S96 antigen, VDS cell-derived mock virus, live N75 virus, and live S96 virus, all added at 100 μl per well. The cells were incubated at 37°C and 5% CO_2_ overnight, the protein transport inhibitor BD GolgiPlug was added to all wells at 1 μl/ml in 30 μl of RPMI 1640, and the cells were incubated for a further 6 h at 37°C and 5% CO_2_. Cells were pelleted at 330 × *g* for 5 min and washed twice with 100 μl of DPBS (Thermo Fisher). The directly conjugated antibodies APC-labeled anti-CD4 and RPE-labeled anti-CD8 were added, along with 0.5 μl per well of Zombie Aqua dead cell stain (BioLegend), and the cells were incubated at 4°C for 30 min in the dark. The cells were again pelleted and washed once in DPBS before fixation with BD Cytofix/Cytoperm buffer (BD Biosciences) containing 4.2% formaldehyde at 4°C for 30 min in the dark. Cells were again pelleted and washed once in DPBS before being stored overnight in 100 μl of DPBS at 4°C. The following morning, the cells were pelleted and permeabilized with 100 μl of BD Perm/Wash buffer (BD Biosciences) for 20 min at 4°C in the dark. Cells were incubated with 100 μl/well anti-IFN-γ conjugated with Alexa Fluor 488 (clone CC302; Bio-Rad Antibodies) diluted 1/320 in BD Perm/Wash Buffer and incubated for 30 min at 4°C in the dark. The cells were washed twice with 150 μl/well BD Perm/Wash buffer before finally being resuspended in 200 μl of MACS wash solution (Miltenyi Biotec). Data were collected with a MACSQuant Analyzer 10 (Miltenyi Biotec) using MACSQuantify software. A sample of 150 μl of the 200 μl per well was analyzed to enable assessment of rare events. Single-labeled cells were used for multicolor compensation for every experiment. All flow cytometry raw data files were analyzed in FlowJo X; the gating strategy used is shown in Fig. 4.

### Detection of PPRV RNA.

RT-qPCR for the detection of PPRV RNA was carried out at the Nonvesicular Reference Laboratory at the Pirbright Institute using the method of Batten et al. (57).

### Construction of phylogenetic tree.

To construct the phylogenetic tree shown in Fig. 1, all the full-length PPRV genomes available in GenBank were assembled and aligned. Where two or more genomes were essentially identical (genetic distance < 0.001), all but one of the sequences were removed from the alignment. The tree was calculated and plotted using MEGA6 (58); the evolutionary history was inferred using the maximum-likelihood method based on the general time reversible model (59) with a discrete gamma distribution to model evolutionary rate differences among sites and a rate variation model which allowed for some sites to be evolutionarily invariable. This model (GTR+G+I) was determined to be the optimal model using jModelTest (60). The final analysis involved 40 nucleotide sequences and 15,948 positions; all positions with less than 80% site coverage were eliminated, that is, fewer than 20% of alignment gaps, missing data, and ambiguous bases were allowed at any position. The reliability of the tree topology was determined by bootstrap using 500 replicates.

### Statistical analysis.

Statistical analysis was carried out using programs available in R (61). Data were analyzed using mixed models in which vaccine and time were fixed factors and individual animals were random factors. Although different batches of vaccination were carried out at different times, the group of vaccinations was not considered a significant factor, since any effects would be included in “animal.” For analysis of cELISA and proliferation data following challenge with wild-type virus, the challenge virus was a third fixed factor. Temperature profiles and clinical score data were analyzed separately for each challenge experiment. Temperature profiles were fit to linear models as fourth order polynomials with respect to time. Clinical scores were compared as the mean score for each animal over the days it was alive to be assessed, using a nonparametric one-way analysis with multiple comparisons, *nparcomp* (62). Linear models were fit using *nlme* (63), and comparisons were carried out using *lsmeans* (64), with appropriate correction for multiple comparisons. In order to improve the normality of the data, virus neutralization titers and T cell proliferation data were analyzed as the log_2_(titer) or log_2_(stimulation index), respectively.
